# Establishment of patient-derived tumor xenograft (PDTX) models using samples from CT-guided percutaneous biopsy

**DOI:** 10.1590/1414-431X20176000

**Published:** 2017-05-18

**Authors:** Y.-P. Zhuang, Y.-P. Zhu, H.-Y. Wang, L. Sun, J. Zhang, Y.-P. Hao, L. Wang

**Affiliations:** 1 Department of Radiology, Jiangsu Cancer Institute and Hospital, Nanjing, Jiangsu, China; 2 Nanjing Personal Oncology Biological Technology Co. Ltd., Nanjing, Jiangsu, China

**Keywords:** Cancer, Patient-derived tumor xenografts (PDTX), Computed tomography, X-ray, Percutaneous biopsy, Mouse models

## Abstract

This study aimed to investigate the feasibility of the establishment of a human cancer xenograft model using samples from computed tomography (CT)-guided percutaneous biopsy. Fresh tumor tissues obtained from 10 cancer patients by CT-guided percutaneous biopsy were subcutaneously inoculated into NOD-Prkdcem26Il2rgem26Nju (NCG) mice to establish human patient-derived tumor xenograft (PDTX) models. The formation of first and second generation xenografts was observed, and tumor volume was recorded over time. Tumor tissue consistency between the PDTX model and primary tumors in patients was compared using H&E staining and immunohistochemistry. Pharmacodynamic tests of clinically used chemotherapeutic drugs were conducted on second generation xenografts, and their effects on tumor growth and body weight were observed. CT-guided percutaneous biopsy samples were successfully collected from 10 patients with advanced cancers. The PDTX model was established in mice using tumor samples obtained from 4 cancer patients, including one small cell carcinoma sample, two adenocarcinoma samples, and one squamous cell carcinoma sample. The success rate was 40%. The obtained PDTX model maintained a degree of differentiation, and morphological and structural characteristics were similar to primary tumors. The pharmacodynamic test of chemotherapeutic drugs in the PDTX model revealed a therapeutic effect on tumor growth, as expected. CT-guided percutaneous biopsy samples can be effectively used to establish a PDTX model, and test these chemotherapy regimens.

## Introduction

The tumor animal model is an effective and commonly used tool for predicting pre-clinical efficacy and evaluating the possible toxicity of anti-tumor drugs. Recently, tumor tissues from surgical ablation have been increasingly used to reconstruct the patient-derived tumor xenografts (PDTX) model by transplantation into immune-deficient mice. This type of model is able to maintain the growth environment of tumor cells, preserve the tumor intercellular matrix and fibroblast cells, retain tumor shapes and vascular network structures, and keep the entire three-dimensional structures of the model tumor, and drug response features of tumor tissues, thereby mimicking the patient's primary tumor with high consistency. For example, researchers at the MD Anderson Cancer Center (1) established PDTX models using fresh lung cancer tissues from 81 patients with non-small cell lung cancer. As a result, models were successfully established in 23 cases, and were found to exhibit pathological changes consistent with the primary tumor. Genetic analysis detected 315 mutations in primary tumor tissues obtained from the patients, and 293 mutations in PDTX models, with a consistency rate of 93%. This indicates that the PDTX model was able to retain mutations in the primary tumor. DeRose et al. (2) successfully established PDTX models using tumor tissues of patients with breast cancer in immune-deficient mice (NOD/SCID). Pathological and immunohistochemical analysis revealed that the pathological type, expression level of molecular markers (cytokeratin protein, E-cadherin, β-catenin, and vimentin), as well as the gene expression of the model tumor tissues were highly consistent with the original samples. Moreover, the growth rate of the triple negative breast cancer model was found to be significantly higher than other types of breast cancer models, which was consistent with clinical results. Hence, the PDTX model is an almost perfect "substitute" for the clinical tumor of a patient.

At present, tissues used for establishing PDTX models have been mainly obtained from resected tumor tissues. However, many advanced cancer patients are no longer suitable for surgery. In such circumstances, the establishment of PDTX models is limited due to the unavailability of tumor tissues from surgery. To date, computed tomography (CT)-guided percutaneous lung biopsy has become the main means for diagnosing lung diseases with high accuracy (3). The advantages of CT-guided biopsy include minimal invasiveness, safety, accurate localization, simple operation, low cost, fewer complications, and suitability for advanced cancer patients. The obtained biopsy samples can be used not only to establish tumor pathological classification and phenotyping, but also to detect tumor-derived gene mutation and screen a population for clinical treatment with targeting drugs available with the development of molecular biotechnology (4,5).

In this study, we attempted to establish PDTX models using tumor tissue samples obtained by CT-guided percutaneous biopsy from cancer patients and determine the adequacy of the samples. Pharmacodynamic tests of chemotherapeutic drugs were also performed.

## Material and Methods

### Experimental animals

A total of 190 SPF-grade 6–8 week-old NCG female mice weighing 20±2 g were purchased from Nanjing University Model Animal Institute. NCG mice, which were used to knockout *Prkdc* and *IL2RG* genes directly in NOD/ShiLtJNju mice using CRISPR/Cas9 technology, can be used for the transplantation of human hematopoietic stem cells, islets and tumor cells. Animals were housed in standard animal facilities with 12-h on-off light conditions, and were allowed free access to standard food and water. The animals were allowed to adapt to their surroundings for at least three days before the experiments. All interventions and animal care procedures were performed in accordance with the Guidelines and Policies for Animal Surgery provided by our hospital, and were approved by the Institutional Animal Use and Care Committee.

### Tumor sample source

*Patients.* From March to June 2015, 10 patients (9 males, 1 female; average age 64 years, range 46–78 years) underwent CT-guided percutaneous biopsy in our institute. Five patients had lesions in the right lung, 4 patients had lesions in the upper lobe of the left lung, and 1 patient had lesions in the liver. The maximum transverse diameter of the lesions ranged between 10–80 mm, with an average diameter of 29 mm. All patients underwent CT plain scan and contrast-enhanced scan before percutaneous biopsy. Clinical symptoms of patients with pulmonary lesions were mainly cough, hemoptysis, chest pain and chest tightness. The patient with lesion in the liver had a symptom of abdominal pain. Patients had no severe cardiac and pulmonary dysfunction, bleeding tendency or bleeding disease, lung congestion, pulmonary artery hypertension, severe pulmonary bullae, and severe emphysema.

*CT-guided biopsy*. CT-guided biopsy was performed under the guidance of 2-row helical CT (Hispeed NX/i; GE Healthcare, USA). The appropriate positions were selected according to lesion location. Scanning range was set to cover the whole lesion. CT scanning parameters were as follows: tube voltage, 120 kV; tube current, 220 mAs; slice thickness, 5 mm; pitch, 1.5. The puncture points were determined using the metal marker positioning method. The rib and scapula were avoided. Biopsy sites were selected on the solid part of the tumors according to their CT enhancement. A 17G cutting needle or 18G Chiba biopsy needle (Precisa, Italy) was used with the connection of a 50-mL syringe needle. The distance, depth and angle of puncture were measured. Four different puncture points were selected, and at least three samples of tumor tissue were obtained. One sample was fixed in 10% formalin for pathological examination including conventional H&E staining and immunohistochemistry staining for CD56 and TTF1. The other two samples were processed for PDTX modeling. Plain CT scan was immediately performed after completion of biopsy, in order to determine whether pneumothorax or hemorrhage complications occurred. Patients were observed to determine whether they had chest pain, dyspnea and hemoptysis symptoms. Chest radiography was obtained after 2 h. CT-guided percutaneous biopsy was successfully conducted in all 10 patients. No patient had a pneumothorax and only 1 patient had slight bleeding in the needle tract, but did not receive any further treatment. Biopsy was performed in 5 patients using a 17-gauge automated cutting biopsy needle with 10- or 20-mm notches for core needle biopsy, and the obtained tumor tissue length was 10–20 mm. Biopsy was performed in another 5 patients using an 18-gauge needle for aspiration, in order to collect the tissues. Biopsy results revealed small cell lung cancer in 3 patients, adenocarcinoma in 5 patients, and squamous cell carcinoma in 2 patients (Table 1).

**Table 1.** Biopsy sites, sampling methods and pathological types for the 10 patients with cancer.

No.	Patient	Gender	Age (years)	Puncture site	Sampling method	Pathological type
1	010000*	Male	64	Lung	Cutting puncture	Small cell carcinoma
2	010024	Male	64	Lung	Cutting puncture	Squamous cell carcinoma
3	010031	Male	46	Lung	Cutting puncture	Small cell carcinoma
4	010034	Male	55	Lung	Cutting puncture	Adenocarcinoma
5	010027*	Male	60	Liver	Cutting puncture	Adenocarcinoma
6	010023	Male	52	Lung	Needle aspiration	Adenocarcinoma
7	010026*	Male	78	Lung	Needle aspiration	Small cell carcinoma
8	010028	Female	76	Lung	Needle aspiration	Adenocarcinoma
9	010032*	Male	74	Lung	Needle aspiration	Squamous cell carcinoma
10	010033	Male	68	Lung	Needle aspiration	Adenocarcinoma

Asterisks indicate successfully established PDTX.

### Tumor xenograft

The obtained fresh tumor tissues were immediately put in Leibowitz's L15 tissue preservation solution containing L-glutamine, 10% neutral formaldehyde and double antibiotics; samples were transferred to Nanjing University Model Animal Research Institute/National Genetic Engineering Resources Laboratory for storage at –80°C. Tumor inoculation in mice was completed within 4 h after sampling. Before implantation, the necrotic tissues of samples were removed, and blood was washed. Solid tumor tissues were selected and cut into 2×2×2 mm pieces, and injected subcutaneously in both sides of the forelimbs. After injection, the needle was slowly withdrawn. Tumor samples of each patient were inoculated into 3–5 mice according to the tissue sample size. After implantation, animals were weighed twice a week. The length and width of the xenografts were measured twice per week after tumor formation, and relative tumor volume was calculated using the formula: relative tumor volume = 0.5×length diameter×short diameter^2^. When first generation tumors (P1) grew to 500 mm^3^, the P1 tumors were removed and implanted into animals using the same manner to produce the second generation tumors (P2). When the P2 tumors grew to 100–150 mm^3^, pharmacodynamic tests were conducted.

### Pharmacodynamics test

Chemotherapy pharmacodynamic tests were conducted on animals bearing the P2 tumors. When tumor size reached 100–150 mm^3^, the animals were randomly assigned to receive chemotherapy treatment according to the histologic type of the primary tumor. For small cell lung cancer (010000), 24 mice received intraperitoneal injections of docetaxel at a dose of 150 mg/kg weekly (n=6), irinotecan at a dose of 100 mg/kg (n=6), or gemcitabine at a dose of 120 mg/kg (n=6); or vehicle for controls (n=6). For small cell carcinoma (010026), 30 mice (n=6, each) received intraperitoneal injection of cisplatin (3.3 mg/kg) weekly plus VP-16 (20 mg/kg, twice weekly), irinotecan (100 mg/kg) weekly, taxol (20 mg/kg) weekly, cisplatin (3.3 mg/kg) plus gemcitabine (80 mg/kg) twice weekly, or vehicle as controls. For adenocarcinoma (010027), 24 mice (n=6, each) received intraperitoneal injection of oxaliplatin (6 mg/kg) every three days, 5-Fu (40 mg/kg) twice weekly, gemcitabine (120 mg/kg) twice weekly, or vehicle as controls. The tumor volume in each group was recorded twice a week, and mice body weight was measured for three weeks. The tumor volume growth curve was recorded.

### Histology

The primary tumor samples of the patients and animal xenografts were sent to the Pathology Department of Jiangsu Provincial Cancer Hospital for pathological examination. The P2 xenografts were removed at three weeks after treatment and processed for the same histological tests as the primary tumors, including H&E staining and immunohistochemistry staining for CD56 and TTF1.

## Results

### PDTX model

All animals recovered well 2 days after inoculation. Animals that received the primary tumor tissues presented round or oval, slightly hard solid nodules bulging from the skin at the inoculation site at 4–6 weeks after tumor tissue implantation. Then, P1 tumor size increased rapidly. In animals that received P1 tumor tissues, P2 tumor volume reached 100–150 mm^3^ at approximately three weeks after inoculation.

**Figure 1.**
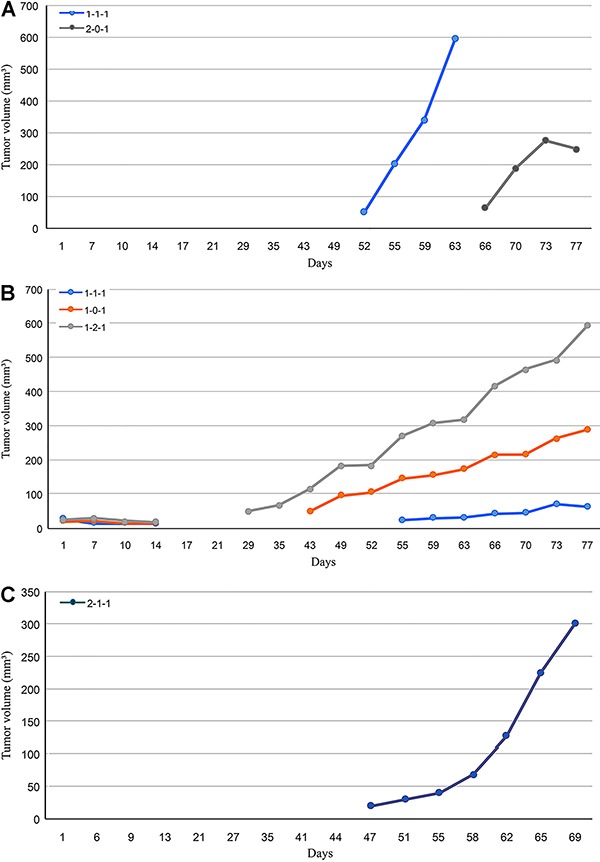
Representative tumor volume (mm^3^) growth curve of an animal with two P1 patient-derived tumor xenograft (PDTX) tumors established from 010026 lung cancer (*A*), an animal with three P1 PDTX tumors from 010027 liver cancer (*B*), and an animal with one P1 PDTX tumor from 010032 lung cancer (*C*).

PDTX models were successfully established using primary tumor tissues from 4 (out of 10) patients including 1 small cell carcinoma patient, 2 adenocarcinoma patients and 1 squamous cell carcinoma patient, giving a success rate of 40%. Among these 4 patients, tumor tissues were obtained through excisional biopsy from 2 patients, and by aspiration biopsy from the other 2 patients. The growth curve of the P1 tumors is shown in Figure 1. The PDTX P2 tumor was successfully established for small cell carcinoma and adenocarcinomas. The procedure failed for squamous cell carcinoma due to prominent tumor necrosis when P1 generation tumor was passaged to P2 generation. Moreover, liver metastasis occurred in one animal with PDTX P2 small cell carcinoma.

### Histology

**Figure 2.**
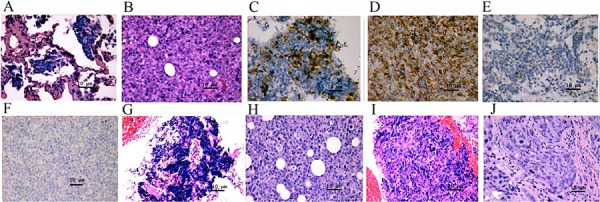
Histology of the primary tumors of patients and patient-derived tumor xenograft (PDTX) tumors. Microphotographs of H&E staining of 010000 in a patient show that the primary tumor (*A*) and PDTX tumor (*B*) exhibited the same cell morphologies. Both the primary tumor (*C*) and PDTX tumor (*D*) were positive for CD56 staining and negative for TTF1 staining (*E* and *F*). Microphotographs of the H&E staining (magnification, ×40) of 010026 in a patient shows that the primary tumor (*G*) and PDTX tumor (*H*) exhibited the same cell morphologies. Microphotographs of the H&E staining of 010027 in a patient show that the primary tumor (*I*) and PDTX tumor (*J*) exhibited the same cell morphologies.

**Figure 3.**
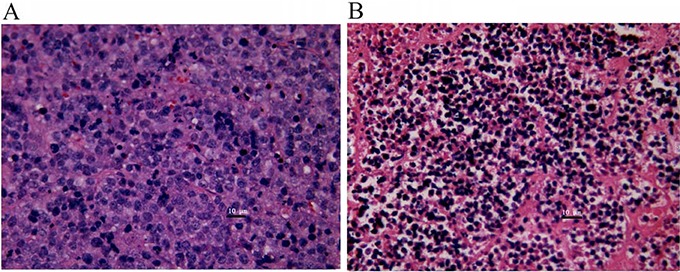
Histology of liver metastasis from one P2 patient-derived tumor xenograft (PDTX) tumor. Microphotographs of H&E staining (magnification ×40) in 1 animal revealed that metastatic lesions in the liver (*A*) and its PDTX P2 tumor (*B*) had the same cell morphology. Both are small cell carcinoma.

The P1 or P2 PDTX models and patients’ primary tumors exhibited a similar degree of differentiation, morphology and structural characteristic, as well as identical immunochemistry staining features (Figure 2). PDTX P2 small cell carcinoma and liver metastasis also had a similar histology (Figure 3).

### Pharmacodynamic test

**Figure 4.**
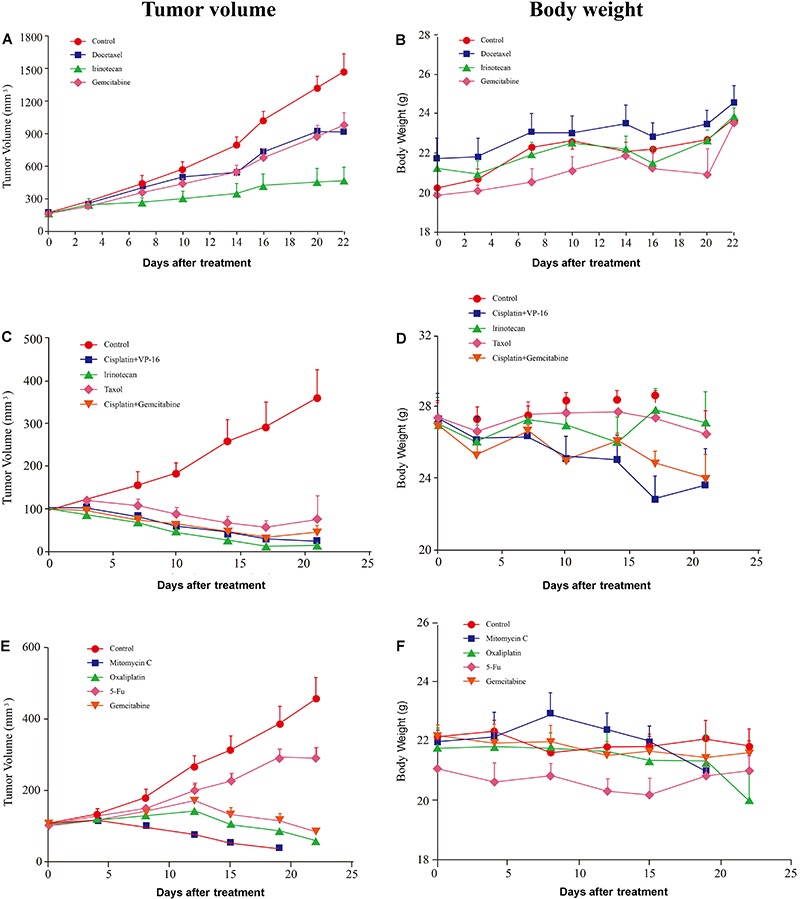
P2 tumor volume/time growth curves and mice body weight/time curves after the administration of chemotherapeutic drugs of patient-derived tumor xenograft (PDTX) small cell carcinoma (010000) (*A* and *B*), lung cancer (010026) (*C* and *D*) and adenocarcinoma (010027) (*E* and F).

The tumor growth and body weight curves in mice bearing P2 tumors are shown in Figure 4. Different chemotherapy regimens exerted different effects on tumor growth. There was no significant change in body weight, and no side effects.

## Discussion

At present, the incidence of cancer is gradually increasing. Since the onset of cancer is usually latent, the disease is often at the metastatic phase or advanced stage when diagnosed; and mortality remains high. The current treatment for cancers remains principally based on surgery, radiotherapy and chemotherapy. At present, the overall efficacy of chemotherapy is approximately 30%. Seventy percent of patients not only fail to achieve the desired treatment effect, but also suffer side effects of chemotherapy. Therefore, it is very important for cancer patients that an effective individualized therapy drug or regimen is determined in a timely manner. The PDTX model is a novel and important platform that meets this urgent need (6).

Previously, it has been shown that the PDTX models of gastric cancer, liver cancer, lung cancer, colorectal cancer, melanin tumor and esophageal cancer maintain the biological characteristics of primary tumors and are, therefore, a stable and reliable animal model for cancer research (1,). Our study provides an important and highly consistent *in vivo* model for the drug treatment of tumors.

In this study, pharmacodynamic tests were conducted in three established PDTX models. Results revealed that the effect of irinotecan was adequate, but the effects of docetaxel and gemcitabine were not obvious for small cell carcinoma. Furthermore, etoposide plus cisplatin, cisplatin plus gemcitabine, paclitaxel, and irinotecan also had adequate effects, but cisplatin plus etoposide had high toxicity for small cell carcinoma. Hepatic adenocarcinoma tests revealed that the effect of mitomycin C was the best, the effect of 5-FU was poor, and oxaliplatin and gemcitabine had certain therapeutic effects. The entire process (from implantation to testing) typically takes 2–4 months. Zhang et al. used the PDTX model to test the antitumor efficacy of gefitinib on non-small cell lung cancer patients with *EGFR*, *KRAS* and *FGFR* gene mutations, and results were consistent with previous clinical trials (11). These findings suggest that the PDTX model is useful for selecting the best individualized treatment, in order to avoid unnecessary medication, reduce toxicity and side effects, improve the life of patients, and prolong survival time.

The tumor tissues used for PDTX modeling were derived mostly from surgical samples. For aggressive tumors that cannot be ablated such as aggressive lung cancer, CT-guided percutaneous lung puncture has become the main means of diagnosis, because it can obtain adequate cells or tissue samples, and has high diagnostic accuracy (3,12). Furthermore, it has been proven that the pathological immunohistochemistry and expression of biological markers between biopsy samples and surgical samples are well correlated (7 [Bibr B08] [Bibr B09] [Bibr B10],^13^–^15^ [Bibr B16]). In the past 10 years, multi-gene detection analysis has been done using biopsy samples, which provide important gene phenotyping decision-making evidence for the use of treatment drugs such as molecular-targeted drugs (14–17). The success rate of PDTX modeling using surgical samples has been reported to be 25–40% (11,18,19). Our success rate of 40% was comparable with surgical samples. Furthermore, it was reported that the success rate of PDTX in small cell lung cancer using ultrasound-guided transbronchial needle aspiration biopsy reached as high as 75% (20). Collectively, these results suggest that biopsy samples can meet the requirements of PDTX modeling. In our study, successful PDTX models were developed with tissues obtained by excisional biopsy and needle aspiration methods. Needle aspiration biopsy can be useful in some high-risk lesions such as hilar lesions, which may not be suitable for excisional biopsy.

In our study, none of the 10 cases of percutaneous biopsy had serious complications. Although the number of patients was small, the common complications caused by puncture such as pneumothorax and hemorrhage were within the clinically acceptable range. It has been shown that increasing the quantity of percutaneous biopsy samples does not increase complications (3,12,21,22). This indicates that CT-guided biopsy is clinically safe even for patients who must receive multiple punctures to ensure that adequate tumor samples are attained for PDTX modeling.

In conclusion, CT-guided percutaneous biopsy of tumor tissue samples can be effectively used for PDTX modeling in patients who are unable to undergo surgery and patients with aggressive cancer. The established PDTX model can be used to select the best individualized treatment regimen, and thereby be used as an important reference for clinical cancer treatment.
